# Domestic violence and perinatal outcomes – a prospective cohort study from Nepal

**DOI:** 10.1186/s12889-019-6967-y

**Published:** 2019-05-31

**Authors:** Kunta Devi Pun, Poonam Rishal, Elisabeth Darj, Jennifer Jean Infanti, Shrinkhala Shrestha, Mirjam Lukasse, Berit Schei, On Behalf Of The ADVANCE Study Group, Ragnhild Lund, Rajendra Koju, Kumudu Wijewardene, Dinusha Chamanie Perera, Munas M. Muzrif, Katarina Swahnberg, Jacquelyn C. Campbell

**Affiliations:** 1 Department of Public Health and Nursing, Faculty of Medicine and Health Sciences, NTNU, Norwegian University of Science and Technology, Trondheim, Norway; 2 Kathmandu University School of Medical Sciences, GPO 11008, Kathmandu, Kavre, Dhulikhel, Nepal; 3 Department of Community Medicine, Kathmandu Medical College and Teaching Hospital, Kathmandu, Nepal; 4 Department of Obstetrics and Gynecology, St. Olavs University Hospital, Trondheim, Norway; 5 Department of Women’s and Children’s Health, Uppsala University, Uppsala, Sweden; 6 Faculty of Health Sciences, Oslo Metropolitan University, Oslo, Norway; 7 Department of Health and Social Sciences, University of Southeast Norway, Oslo, Norway

**Keywords:** Domestic violence, Perinatal outcomes, Low birthweight, Preterm birth, Cesarean section

## Abstract

**Background:**

Domestic violence is one of the most common forms of violence against women. Domestic violence during pregnancy is associated with adverse perinatal and maternal outcomes. We aimed to assess whether domestic violence was associated with mode of delivery, low birthweight and preterm birth in two sites in Nepal.

**Methods:**

In this prospective cohort study we consecutively recruited 2004 pregnant women during antenatal care at two hospitals between June 2015 and September 2016. The Abuse Assessment Screen (modified) was used to assess fear and violence. Having ever experienced either fear or violence was defined as any domestic violence. Obstetric outcomes were obtained from hospital records for 1381 (69%) women, selecting singleton pregnancies only. Mode of delivery was assessed as birth by cesarean section or not. A birthweight of less than 2500 g was defined as low birthweight and preterm birth as birth before completion of 37 weeks gestation. Descriptive and multiple logistic regression analyses were performed to assess associations.

**Results:**

Twenty percent of the women reported any domestic violence. Among all 1381 women, 37.6% gave birth by cesarean section. Of those women who delivered by cesarean section, 84.7% had an emergency cesarean section. Less than 10% of the babies were born prematurely and 13.5% were born with low birthweight. We found no significant association between exposure to any domestic violence during pregnancy and risk of a low birthweight baby or birth by cesarean section. However, having experienced both violence and fear was significantly associated with giving birth to a preterm infant [aOR 2.33 (95% CI;1.10–4.73)].

**Conclusions:**

Domestic violence is common in Nepal. This is a potential risk factor for severe morbidity and mortality in newborns. We found that the risk of having a preterm baby was higher for pregnant women who experienced both fear and violence. This should be recognized by the health sector. In this study, no significant differences were found in the rate of cesarean section nor low birthweight for women who had experienced any domestic violence compared to those who did not.

**Electronic supplementary material:**

The online version of this article (10.1186/s12889-019-6967-y) contains supplementary material, which is available to authorized users.

## Background

Domestic violence (DV) is one of the most common forms of violence against women [1]. It is defined in Nepal by the Ministry of Law, Justice and Parliamentary Affairs as any form of physical, mental, sexual, or economic harm, including acts of reprimand or emotional harm, perpetrated by one person on another with whom he or she has a family relationship [2]. The term DV is applied in Nepal instead of intimate partner violence (IPV) because women often live in extended families and other family members may be the perpetrators of violence.

In Nepal, women typically relocate to their husband’s homes after marriage and may become vulnerable to various forms of DV in the new household setting [3]. A woman’s submissive role in her new home may generate fear irrespective of violence, mainly due to inequalities and power imbalances between men and women [4]. Women’s fear is considered as emotional violence perpetrated by her husband or other family members [5].

The five-item Abuse Assessment Screen (AAS) has been widely used internationally to identify DV during pregnancy [6], including in low-income populations and with uninsured women in Brazil and Sri Lanka [7]. One of the questions in the AAS assesses ‘fear’. Women are asked ‘are you afraid of someone in the family?’ The other questions assess if they have ever been emotionally, physically or sexually abused. A positive answer to any of the AAS questions defines a woman as having experienced any form of DV [5].

Since 2002, DV has been on the health agenda in Nepal and recognized as a risk factor for adverse pregnancy and childbirth outcomes [8]. Direct and/or indirect pathways lead from DV to adverse health outcomes [9]. Direct violence can result in physical injury and/or death [9]. Examples of indirect effects of DV are delayed or no health consultations and stress [9]. Stress during pregnancy raises cortisol levels which can lead to constriction of the blood vessels, limiting blood flow to the uterus and resulting in reduced blood supply to the unborn child [10, 11]. This in turn may cause low birthweight (LBW) [11]. A further consequence of stress during pregnancy may be preterm contractions and preterm birth (PTB) [10].

LBW (< 2500 g at birth) is an important determinant of infant survival and development [12]. In 2010, the highest regional proportion of LBW babies (26%) was recorded in South Asia [13]. In Nepal, the proportion of infants born with LBW was 12.4% in 2011 [14] . LWB may be the result of intrauterine growth restriction or preterm birth. Preterm birth (PTB) is defined as being born before 37 weeks of gestational age. It may be spontaneous and the result of infections and/or premature rupture of membranes, or provider-initiated through induction or cesarean section (CS) [15]. Worldwide estimates of PTB range from 9% in high income countries to 11.8% in low income countries [16].

Increased operative interventions have also been associated with DV. Women exposed to DV are at increased risk of giving birth by Cesarean Section (CS) according to European studies [17, 18]. Women reporting current suffering from adult sexual abuse had the highest risk of an elective CS [17]. There are large variations in CS rates within countries, depending on the type of health facility and sector (public or private). In Nepal, CS estimates range from 1.6 to 49.5% [19].

DV has been increasingly acknowledged as a concern within the scope of antenatal care, and a range of studies have assessed its potential detrimental health effects on pregnant women and their newborns [20, 21]. Only a few studies have been conducted in low and middle income countries (LMICs) on DV and perinatal outcomes and the results are inconclusive [22–25]. In Nepal, no prior studies have explored this topic. The aim of our study was to assess whether DV was associated to mode of delivery and explore the relationships between DV and LBW and PTB, in a prospective cohort of non-selected pregnant women.

## Methods

We performed a prospective cohort study in which we followed singleton pregnant women recruited from two hospitals in Nepal – Dhulikhel Hospital (DH) and Kathmandu Medical College and Teaching Hospital (KMC). DH is a community-based non-profit tertiary care institution situated 30 km east of Kathmandu and KMC is a public-private referral hospital in Kathmandu city.

Pregnant women between 12 to 28 weeks of gestational age were consecutively recruited to the study in both sites during antenatal care [26]. Women with disabilities in vision and hearing, severe illness, in need of emergency assistance, or who did not understand Nepali, were excluded. A Color-Coded Audio Computer-Assisted Self-Interview (C-ACASI) was used wherein women answered a questionnaire in private on a tablet computer [26]. This allowed us to obtain information directly and confidentially from women about DV, sociodemographic and obstetric characteristics, and anxiety and depression. Information on birth outcomes was retrieved from the women’s delivery records at DH and KMC. The extracted data was entered manually into a form developed in Open Data Kit (ODK) by trained research assistants and the first and second authors, between June 2015 and September 2016.

Of the 2004 women included at baseline, corresponding delivery records were identified for a total of 1382 (69%) of the women (DH 681, KMC 701). One woman from KMC was excluded due to twin delivery. Thus among 2003 women, 1381 had given birth at the two hospitals. Details of the recruitment process into the baseline study are published elsewhere [26].

### Variables

The information on background characteristics and DV was collected at baseline using a modified version of the five-item Abuse Assessment Screen (AAS). A detailed description of the AAS, including our modifications to it, is presented elsewhere [26]. The women were asked to indicate whether they were ever afraid of anyone in their family, had experienced emotional and physical violence in their lifetime, physical and sexual violence before pregnancy and physical violence in their current pregnancy. Women with an affirmative response to any of the five AAS screening questions for DV were classified as having experience of ‘any form of DV’ and those with a negative responses were classified as having ‘no exposure’. Sub-categories were created within the group of women having experience of any DV. Women who reported they feared someone in the family but gave negative responses to any other experience of DV were classified as having experienced ‘fear only’. In Nepal, ‘fear’ for women might either be related to power imbalances in her new home after marriage or due to DV [26]. Women who responded affirmatively to any physical, emotional or sexual violence, but not to fear, were classified as being exposed to ‘violence only’. Women with affirmative responses to the questions on fear and violence were classified as having experience of ‘both fear and violence’.

The sociodemographic characteristics included age, education and income of the woman and her husband and, for women only, their caste, ethnicity, family structure, geographical setting, knowledge of a financial incentive to give birth in health institutions, and permission to use their own income.

Symptoms of anxiety and depression were assessed with the Hopkins Symptom Checklist-5 (HSCL-5) which includes feeling fearful, nervousness or shakiness, feeling hopeless about the future, feeling blue, and worrying too much about things. Women were asked to indicate the severity of their feelings on a scale from one (‘not at all’) to four (‘extremely’). Total mean score was calculated following guidance for use of this short version of the scale, and women who scored above two were defined as suffering from mental distress [27].

The outcome data collected from the medical records included mode of delivery and the status of the newborn: live birth or not, gestational age and weight, Apgar score at five minutes after birth, and whether the newborn was transferred to the neonatal intensive care unit (NICU). Infants weighing less than 2500 g were defined as being born with LBW. PTB was defined as a delivery before 37 completed weeks of gestation.

Women whose mode of delivery was by CS were compared to women who gave birth vaginally; either spontaneous or instrumental. Indications of CS were classified as prolonged labour, breech presentation, cephalo-pelvic disproportion, other fetal causes including fetal distress and intrauterine growth restriction or maternal causes such as maternal distress, antepartum hemorrhage, etc., and previous CS. For some women, information was missing on the indication for CS and they were classified as unknown.

### Statistical analysis

Descriptive analyses were performed using SPSS software version 24 to assess and compare the socio-economic and obstetric characteristics of the women between exposed and unexposed. We also assessed if there were significant differences between the women with and without delivery records. The women’s obstetric characteristics were compared between the two hospitals. To estimate associations between DV reported in pregnancy and risks of having LBW, PTB and birth by CS, crude and multiple logistic regression analyses were performed in two models. Based on prior studies, we adjusted for study site, age, education and parity in the first model. Additional adjustments for geographical setting and family type were performed in the second model. Finally, we performed stratified regression analyses by parity adjusting for the same co-variates in both models except for parity. Confidence intervals (CI) were calculated at the 95% level.

## Results

Birth outcomes were obtained for 69% (*n* = 1381) of the women recruited to the study at baseline. The women for whom no delivery records were found did not differ significantly from the women who gave birth at the hospitals in terms of their DV status, sociodemographic characteristics, baseline symptoms of anxiety and depression, or parity (Additional file 1). A total of 283 women (20.5%) reported having experience of any form of DV. A total of 235 (17%), reported being afraid of someone in the family at the time of completing the questionnaire, while 5.2% reported having experienced emotional or physical abuse combined, 2.5% physical abuse only, 1.5% physical abuse during the current pregnancy, and 0.9% had experienced sexual abuse (Additional file 3). The women reporting any DV were significantly younger, less educated and had less autonomy over their income. They were also more often from rural areas, of Dalit (the most oppressed social class) and Janajatis, and reported more symptoms of anxiety and depression at baseline (Table 1).

**Table 1:** Socio-economic characteristics of women by any domestic violence in two hospitals in Nepal, 2016

Characteristics	Total		Any domestic violence				*p* value
			No		Yes		
	*N* = 1381		*n* = 1098		*n* = 283		
	*n*	%	n	%	*n*	%	
Study site (*n* = 1381)							
Dhulikhel Hospital	681	49.3	529	48.2	152	53.7	0.056
Kathmandu Medical College	700	50.7	569	51.8	131	46.3	
Woman’s age in years (*n* = 1381)							
15–19	77	5.6	59	5.4	18	6.4	0.029
20–24	591	42.8	451	41.1	140	49.5	
25–29	519	37.6	423	38.5	96	33.9	
≥ 30	194	14.0	165	15.0	29	10.2	
Woman’s education (*n* = 1379)							
None	131	9.5	90	8.2	41	14.5	< 0.001
Primary	190	13.8	136	12.4	54	19.1	
Secondary	317	23.0	242	22.1	75	26.6	
Higher	741	53.7	629	57.3	112	39.7	
Woman’s income (1381)							
No income	1021	73.9	811	73.9	210	74.2	< 0.001
Income no autonomy	84	6.1	53	4.8	31	11.0	
Income and autonomy	276	20.0	234	21.3	42	14.8	
Family structure (*n* = 1332)							
Nuclear	632	47.4	514	48.5	118	43.4	0.075
Extended	700	52.6	546	51.5	154	56.6	
Geographical setting (*n* = 1381)							
Rural	404	29.3	309	28.1	95	33.6	0.044
Urban	977	70.7	789	71.9	188	66.4	
Caste and ethnicity (*n* = 1380)							
Dalit^a^	38	2.8	25	2.3	13	4.6	0.005
Disadvantaged Janajati^b^	298	21.6	224	20.4	74	26.1	
Advantaged Janajati^c^	306	22.2	238	21.7	68	24.0	
Upper caste^d^	738	53.5	610	55.6	128	45.2	
Baseline anxiety and depression (HSCL-5 score^e^)							
≤ 2	1061	76.8	906	82.5	155	54.8	< 0.001
> 2	320	23.2	192	17.5	128	45.2	

^a^Dalit = The most oppressed social class

^b^Disadvantaged Janajati = Indigenous groups with little or no social mobility

^c^Advantaged Janajati = Indigenous groups with opportunity and access to social mobility

^d^Upper castes = Traditionally, the most privileged groups in the social hierarchy

^e^HSCL-5 score: Hopkins Symptom Checklist score-5

Among all 1381 women, the majority of women visited antenatal care (ANC) at least four times. However, a higher proportion (38.2%) of women at KMC had less than four ANC visits compared to DH (4.9%) (Table 2).

**Table 2:** Obstetric characteristics by any domestic violence in two hospitals in Nepal, 2016

Characteristics	Total		Any domestic violence				*p* value
			No		Yes		
	*N* = 1381		*n* = 1098		*n* = 283		
	*n*	%	*n*	%	*n*	%	
Parity							
Nulliparous	705	51.0	571	52.0	134	47.3	0.092
Multiparous	676	49.0	527	48.0	149	52.7	
Antenatal visits before birth (*n* = 1364)							
< 4	294	21.6	235	21.7	59	21.0	0.435
≥ 4	1070	78.4	848	78.3	222	79.0	
Knowledge and access of incentive (*n* = 1379)							
No knowledge	361	26.2	269	24.5	92	32.7	0.003
Knowledge no access	91	6.6	67	6.1	24	8.5	
Knowledge and access	927	67.2	762	69.4	165	58.7	
Mode of delivery (*n* = 1380)							
Vaginal delivery	809	54.6	639	58.2	170	60.1	0.83
Instrumental delivery	52	3.8	41	3.7	11	3.9	
Cesarean section	519	37.6	417	38.0	102	36.0	
Cesarean section (*n* = 518)							
Elective	79	15.3	68	16.3	11	10.8	
Emergency	439	84.7	348	83.7	91	89.2	
Indication of cesarean section (*n* = 388)							
Prolonged labor	59	15.2	51	16.2	8	11.0	0.705
Breech presentation	45	11.6	37	11.7	8	11.0	
Cephalo-pelvic disproportion	20	5.2	16	5.1	4	5.5	
Other fetal causes	73	18.8	61	19.4	12	16.4	
Maternal causes	69	17.8	51	16.2	18	24.7	
Previous cesarean section	107	27.6	87	27.6	20	27.4	
Unknown reasons	15	3.9	12	3.8	3	4.1	
Gestational age at recruitment (*n* = 1322)							
12–24 weeks	1041	78.7	830	78.9	211	78.1	0.423
25–28 weeks	281	21.3	222	21.1	59	21.9	
Gestational age at birth (*n* = 1372)							
< 37 weeks	122	8.9	91	8.4	31	11.0	0.107
≥ 37 weeks	1250	91.1	998	91.6	252	89.0	
Birthweight (*n* = 1353)							
≥ 2500 g	1171	86.5	932	86.8	239	85.7	0.345
< 2500 g	182	13.5	142	13.2	40	14.3	
Live birth							
No	11	0.8	9	0.8	2	0.7	0.601
Yes	1370	99.2	1089	99.2	281	99.3	
Apgar score at five minutes after birth (*n* = 1327)							
< 7	61	4.6	49	4.6	12	4.5	0.529
≥ 7	1266	95.4	1009	95.4	257	95.5	
Admission to neonatal intensive care unit (*n* = 1378)							
No	1067	77.4	837	76.4	230	81.3	0.047
Yes	311	22.6	258	23.6	53	18.7	

Among all women, 37.6% gave birth by CS. Of those women who delivered by CS, 84.7% had an emergency CS. Previous CS (27.6%) was the most common indication for CS. Less than 10% of the newborns were born preterm and 13.5% were born with low birthweight (Table 2).

Women giving birth at DH were significantly less likely to be delivered by CS (28.3%) compared to KMC (46.6%), and more newborns born at DH had a lower birthweight (15.9%) compared to KMC (11.0%) (Additional file 2). There was no significant difference between the two hospitals as to gestational age at birth (Additional file 2).

We found no statistically significant association between DV and mode of delivery, nor between DV and LBW (Table 3). Having reported both violence and fear was significantly associated to PTB after adjustments, aOR 2.27 (1.16–4.79) in model one and aOR 2.33 (1.10–4.73) in model 2, for all women (Table 3). In the stratified analyses this significant relationship was only present for primiparous women (Table 3).

**Table 3:** Associated factors between any domestic violence and low birthweight, preterm birth or cesarean section of Nepal, 2016^a^

Domestic violence	Low birthweight			Preterm birth			Cesarean section		
	cOR (95% CI)	Model 1	Model 2	cOR (95% CI)	Model 1	Model 2	cOR (95% CI)	Model 1	Model 2
		aOR (95% CI)	aOR (95% CI)		aOR (95% CI)	aOR (95% CI)		aOR (95% CI)	aOR (95% CI)
All women									
No DV	Reference	Reference	Reference	Reference	Reference	Reference	Reference	Reference	Reference
Any DV	0.89 (0.60–1.31)	0.98 (0.60–1.34)	0.91 (0.61–1.36)	0.71 (0.46–1.12)	0.71 (0.54–1.10)	0.70 (0.51–1.14)	1.17 (0.82–1.42)	0.99 (0.74–1.32)	0.99 (0.75–1.33)
Fear only	1.29 (0.82–2.04)	1.37 (0.80–2.03)	1.23 (0.77–2.01)	1.25 (0.72–2.26)	1.25 (0.72–2.26)	1.26 (0.72–2.23)	0.83 (0.59–1.18)	0.89 (0.62–1.27)	0.94 (0.63–1.28)
Violence only	1.22 (0.53–2.80)	1.25 (0.54–2.90)	1.3 (0.57–3.02)	1.04 (0.37–2.97)	1.05 (0.45–3.01)	1.04 (0.43–3.00)	1.05 (0.56–1.93)	1.15 (0.61–2.22)	1.12 (0.69–2.11)
Both fear and violence	0.64 (0.25–1.63)	0.61 (0.24–1.64)	0.62 (0.24–1.64)	2.23 (1.12–4.56)	2.27 (1.16–4.79)	2.33 (1.10–4.73)	1.12 (0.66–1.92)	1.35 (0.74–2.23)	1.30 (0.72–2.21)
Primiparous									
No DV	Reference	Reference	Reference	Reference	Reference	Reference	Reference	Reference	
Any DV	1.58 (0.93–2.50)	1.69 (0.91–2.78)	1.53 (0.89–2.64)	1.54 (0.88–2.83)	1.51 (0.81–2.90)	1.52 (0.87–2.94)	0.98 (0.60–1.34)	1.01 (0.70–1.55)	1.00 (0.71–1.54)
Fear only	1.59 (0.86–2.94)	1.75 (0.89–3.11)	1.54 (0.81–2.92)	1.31 (0.63–2.90)	1.34 (0.64–2.83)	1.38 (0.66–2.93)	0.93 (0.67–1.50)	1.00 (0.61–1.71)	1.02 (0.61–1.70)
Violence only	2.05 (0.73–5.75)	2.5 (0.85–7.31)	2.71 (0.92–7.98)	0.64 (0.08–4.63)	0.54 (0.07–4.22)	0.53 (0.07–4.25)	1.21 (0.58–2.83)	1.53 (0.68–3.75)	1.42 (0.56–3.65)
Both	0.75 (0.15–2.90)	0.65 (0.15–2.90)	0.73 (0.16–3.25)	3.41 (1.20–9.65)	4.25 (1.42–12.42)	4.10 (1.70–12.12)	0.62 (0.23–1.53)	0.70 (0.33–1.99)	0.64 (0.24–1.74)
Multiparous									
No DV	Reference	Reference	Reference	Reference	Reference	Reference	Reference	Reference	
Any DV	0.84 (0.52–1.51)	0.86 (0.43–1.43)	0.83 (0.43–1.42)	1.33 (0.74–2.83)	1.31 (0.72–2.49)	1.32 (0.73–2.42)	0.95 (0.65–1.48)	0.99 (0.67–1.50)	0.99 (0.77–1.58)
Fear only	1.02 (0.51–2.03)	0.94 (0.47–1.90)	0.94 (0.53–1.89)	1.2 (0.57–2.61)	1.29 (0.55–2.55)	1.25 (0.56–2.62)	0.74 (0.45–1.22)	0.80 (0.51–1.34)	0.77 (0.51–1.34)
Violence only	0.69 (0.14–2.)61	0.63 (0.14–2.76)	0.64 (0.15–2.82)	1.45 (0.40–4.85)	1.41 (0.40–4.94)	1.43 (0.41–5.02)	0.96 (0.41–2.23)	0.97 (0.41–2.30)	0.96 (0.40–2.35)
Both	0.62 (0.24–2.15)	0.55 (0.16–1.86)	0.54 (0.16–1.84)	1.65 (0.65–4.24)	1.54 (0.57–4.21)	1.56 (0.60–1.80)	1.69 (0.81–3.15)	1.74 (0.98–3.54)	1.74 (0.87–3.50)

Abbreviations: *OR* Odds ratio, *cOR* Crude odds ratio, *aOR* Adjusted odds ratio

Model 1: adjusted for study site, women’s age, education and parity (model not stratified)

Model 2: adjusted for study site, women’s age, education, geographical setting and family type, parity (for the analysis of all women)

^a^among women with complete case information (*N* = 1330)

## Discussion

Women reporting both violence and fear were significantly more likely to give birth before term compared to women who did not report any experience of DV. This relationship remained significant only for primiparous women in the stratified adjusted analysis. We did not find a significant association between any form of DV reported in pregnancy and having a LBW baby or giving birth by CS.

Nine percent of newborns in our study were born preterm, based on the assessment of gestational age by women’s reported last menstrual period, This is in line with 8.1% PTB found in a tertiary hospital of Nepal [28]. Compared to other LMICs, our estimated rate of PTB was slightly higher than in Tanzanian (7.9%) and Vietnamese (2.7%) cohorts [22, 23]. These other studies both used obstetric ultrasound scanning before 24 weeks of gestation to determine gestational age at birth. Our reliance on women’s reported last menstrual period may have yielded a falsely high prevalence rate of PTB due to recall bias as in Brazilian and Canadian studies [24, 29]. At the two sites in our study anomaly scanning is performed but not routine dating scanning. Prior studies have shown particular forms of violence, especially severe forms of physical or sexual violence, to be associated with LBW and PTB [22–24]. In line with the Vietnamese [23] and Tanzanian [22] studies, we found that experiencing both fear and violence was associated to PTB. In our study, having experienced both fear and violence, very likely included the women experiencing more severe violence compared to those reporting fear only or violence only. However, more detailed patient histories may have helped us to identify other subgroups of women.

The prevalence of LBW in our study is 13.5%, aligning with the reported national rate [14] and similar to a Brazilian study where LBW was reported as 13.8% [15]. The proportion of LBW in our study is higher than in other cohort studies in LMICs [22, 23, 30]. This may be due to the low socio-economic status of women in our study, as this factor is one of the strongest predictors of LBW in low-income countries [31]. As in Brazil [30] and Pakistan [25], we also found no association between DV and LBW.

Based on information from 150 countries, 18.6% of all births occur by CS, with the lowest proportion (6%) in low-income countries [32]. In our study, among all births, 37.6% occurred by CS, with CS rates at KMC being significantly higher than at DH. These CS rates are far higher than the national Nepali average of 12.0% (public sector 1.6% and private sector 49.5%) [19]. This may be due to the fact that both hospitals are tertiary level referral hospitals and they neither are government hospitals. Contradictory to two European studies [17, 18], however, women exposed to DV in our study were not at a higher risk of having a CS. The lack of association in our study may be the result of selection biases. It is possible that women who were exposed to DV and had poor perinatal outcomes, delivered elsewhere. We can also hypothesize that controlling behaviors by abusers may have hindered women from returning to the hospitals to receive treatment for pregnancy complications or to give birth. By contrast, it may have been more likely for women with no exposure to DV to be encouraged by their families to seek hospital care when experiencing pregnancy complications. These potential selection biases could have led to an underestimation of the association between DV and poor outcomes.

We might also have misclassified exposed women as non-exposed to any DV. The questions we asked about abuse may have excluded context-specific DV; for example, DV perpetrated by mothers-in-law by depriving daughters-in-law of food, impeding access to pregnancy care or accepting DV as the husbands right to discipline his family [3]. Also, women may have underreported DV on account of its sensitivity and taboo status in Nepal, because women are taught to silently endure DV, or reporting it may have felt like a hindrance to familial reconciliation [3]. Additionally, women may have encountered DV at a later stage in their pregnancies than when our estimates were made between 12 to 28 weeks of gestational age. Any women who experienced DV after this time period would have been classified as non-exposed in our study; hence the lack of associations we found could be due to diluting of effects.

The etiologies of PTB and LBW are multifactorial and we have not taken all factors into account, such as women’s short stature, low body mass index (BMI), smoking (or second-hand smoking by use of low quality household fuel), and alcohol consumption [33]. Likewise, physical work during pregnancy, hemoglobin levels below 11 g/dl, and poor nutritional status during pregnancy were significantly associated with LBW in another study in a tertiary hospital of Nepal [34]. These findings were supported in the recent Nepal Demographic and Health Survey (NDHS) 2016 where 17% of women in reproductive age groups were classified as undernourished with BMIs of < 17 kg/m2 [35]. These confounders may dilute any effect of DV in our study.

Despite the limitations, our study is notable for its inclusion of a large non selective population of pregnant women at two sites. Although we could not identify birth records for all women, we found no significant selection biases in the background variables between the women with and without delivery records. In Nepal, 57% of births take place at a health facility [35]. Many women in Nepal give birth at home. This offers some explanation for why we only found records for 69% of the women attending ANC. The study information was obtained with standardized instruments, and the delivery outcomes were recorded independent of the baseline information. To our knowledge, this is the first prospective cohort study conducted with pregnant women in routine care in Nepal.

## Conclusions

DV is potentially lethal, and ANC provides a window of opportunity to identify exposed women, improve their safety, and provide relevant care. Our study indicates that DV is among the risk factors for preterm delivery, which may lead to severe morbidity and mortality in newborns. To improve the response of health care systems to DV in LMICs, it is important to continue to increase evidence-based knowledge about the effects of DV on pregnant women, as well as the capacity of staff working in ANC to respond to DV, and explore the implementation of interventions.

## Additional files

Additional file 1: Characteristics of women with and without delivery records in Nepal, 2016. (DOCX 25 kb)

Additional file 2: Distribution of obstetric characteristics among women of Nepal, 2016. (DOCX 21 kb)

Additional file 3: Prevalence of domestic violence and low birthweight, preterm birth or cesarean section of Nepal, 2016 (DOCX 16 kb)

## Abbreviations

AAS: Abuse Assessment Screen
ANC: Antenatal care
C-ACASI: Color-Coded Audio Computer-Assisted Self-Interview
CS: Cesarean section
DH: Dhulikhel Hospital
DV: Domestic violence
HSCL-5: Hopkins Symptom Checklist-5
IPV: Intimate partner violence
KMC: Kathmandu Medical College and Teaching Hospital
LBW: Low birthweight
LMIC: Low and middle income countries
NICU: Neonatal Intensive Care Unit
ODK: Open data kit
PTB: Preterm birth

## References

[1] Heise L, Ellsberg M, Gottemoeller M (1999). Ending violence against women. Popul Rep.

[2] Ministry of Law and Justice (2009). Domestic violence (offence and punishment) act, 2066.

[3] Pun KD, Infanti JJ, Koju R, Schei B, Darj E, ADVANCE Study Group. Community perceptions on domestic violence against pregnant women in Nepal: a qualitative study. Glob Health Action. 2016;9:31964. pmid27882865. 10.3402/gha.v9.31964 PMC5122230 27882865

[4] Emery CR, Thapa S, Wu S (2017). Power and control in Kathmandu: a comparison of attempted power, actual power, and achieved power. Violence Against Women.

[5] Hammoury N, Khawaja M (2007). Screening for domestic violence during pregnancy in an antenatal clinic in Lebanon. Eur J Pub Health.

[6] Basile KC, Hertz MF, Back SE (2007). Intimate partner violence and sexual violence victimization assessment instruments for use in healthcare settings.

[7] Rabin RF, Jennings JM, Campbell JC, Bair-Merritt MH (2009). Intimate partner violence screening tools: a systematic review. Am J Prev Med.

[8] Colombini Manuela, Mayhew Susannah H, Hawkins Ben, Bista Meera, Joshi Sunil Kumar, Schei Berit, Watts Charlotte (2015). Agenda setting and framing of gender-based violence in Nepal: how it became a health issue. Health Policy and Planning.

[9] García-Moreno C, Pallitto C, Devries K, Stöckl H, Watts C, Abrahams N. Global and regional estimates of violence against women: prevalence and health effects of intimate partner violence and non-partner sexual violence: World Health Organization; 2013.

[10] Wadhwa PD, Entringer S, Buss C, Lu MC (2011). The contribution of maternal stress to preterm birth: issues and considerations. Clin Perinatol.

[11] Altarac M, Strobino D (2002). Abuse during pregnancy and stress because of abuse during pregnancy and birthweight. JAMWA..

[12] Wilcox AJ (2001). On the importance—and the unimportance—of birthweight. Int J Epidemiol.

[13] Lee AC, Katz J, Blencowe H, Cousens S, Kozuki N, Vogel JP (2013). National and regional estimates of term and preterm babies born small for gestational age in 138 low-income and middle-income countries in 2010. Lancet Glob Health.

[14] Ministry of Health and Population (MOHP) (2012). New Era, and ICF International Inc. Nepal demographic and health survey 2011.

[15] Audi CAF, Corrêa AMS, MdRDd L, Santiago SM (2008). The association between domestic violence during pregnancy and low birth weight or prematurity. J Pediatr.

[16] Blencowe H, Cousens S, Oestergaard MZ, Chou D, Moller A-B, Narwal R (2012). National, regional, and worldwide estimates of preterm birth rates in the year 2010 with time trends since 1990 for selected countries: a systematic analysis and implications. Lancet.

[17] Schei B, Lukasse M, Ryding EL, Campbell J, Karro H, Kristjansdottir H (2014). A history of abuse and operative delivery--results from a European multi-country cohort study. PLoS One.

[18] Tomasdottir MO, Kristjansdottir H, Bjornsdottir A, Getz L, Steingrimsdottir T, Olafsdottir OA (2016). History of violence and subjective health of mother and child. Scand J Prim Health Care.

[19] Ministry of Health (2017). New ERA, Nepal Health Sector Support Program (NHSSP), Inner City fund (ICF). Nepal health facility survey 2015.

[20] World Health Organization (WHO), Department of Reproductive Health and Research, London School of Hygiene and Tropical Medicine, South African Medical Research Council (2013). Global and regional estimates of violence against women:prevalence and health effects of intimate partner violence and non-partner sexual violence.

[21] World Health Organization. WHO multi-country study on women's health and domestic violence against women: summary report of initial results on prevalence, health outcomes and women's responses. 2005.

[22] Sigalla GN, Mushi D, Meyrowitsch DW, Manongi R, Rogathi JJ, Gammeltoft T (2017). Intimate partner violence during pregnancy and its association with preterm birth and low birth weight in Tanzania: a prospective cohort study. PLoS One.

[23] Hoang TN, Van TN, Gammeltoft T, Meyrowitsch DW, Thuy HNT, Rasch V (2016). Association between intimate partner violence during pregnancy and adverse pregnancy outcomes in Vietnam: a prospective cohort study. PLoS One.

[24] Nunes MAA, Camey S, Ferri CP, Manzolli P, Manenti CN, Schmidt MI (2011). Violence during pregnancy and newborn outcomes: a cohort study in a disadvantaged population in Brazil. J Public Health.

[25] Zareen N, Majid N, Naqvi S, Saboohi S, Fatima H (2009). Effect of domestic violence on pregnancy outcome. J Coll Physicians Surg Pak.

[26] Rishal Poonam, Pun Kunta Devi, Darj Elisabeth, Joshi Sunil Kumar, Bjørngaard Johan Håkon, Swahnberg Katarina, Schei Berit, Lukasse Mirjam, Schei Berit, Bjørngaard Johan Håkon, Darj Elisabeth, Infanti Jennifer J., Lund Raghnild, Lukasse Mirjam, Swahnberg Katarina, Campbell Jacquelyn C., Joshi Sunil Kumar, Koju Rajendra, Pun Kunta Devi, Wihewardene Kumudu, Perera Dinusha Chamanie, Muzrif Mohamed Munas Mohamed (2017). Prevalence and associated factors of domestic violence among pregnant women attending routine antenatal care in Nepal. Scandinavian Journal of Public Health.

[27] Strand BH, Dalgard OS, Tambs K, Rognerud M (2003). Measuring the mental health status of the Norwegian population: a comparison of the instruments SCL-25, SCL-10, SCL-5 and MHI-5 (SF-36). Nord J Psychiatry.

[28] Kc A, Wrammert J, Nelin V, Ewald U, Clark R, Malqvist M (2015). Level of mortality risk for babies born preterm or with a small weight for gestation in a tertiary hospital of Nepal. BMC Public Health.

[29] Urquia ML, O'Campo PJ, Heaman MI, Janssen PA, Thiessen KR (2011). Experiences of violence before and during pregnancy and adverse pregnancy outcomes: an analysis of the Canadian maternity experiences survey. BMC Pregnancy Childbirth.

[30] Rodrigues DP, Gomes-Sponholz FA, Stefanelo J, Nakano AMS (2014). Monteiro JCdS. Intimate partner violence against pregnant women: study about the repercussions on the obstetric and neonatal results. Revista da Escola de Enfermagem da USP.

[31] Mumbare SS, Maindarkar G, Darade R, Yenge S, Tolani MK, Patole K (2012). Maternal risk factors associated with term low birth weight neonates: a matched-pair case control study. Indian Pediatr.

[32] Betrán AP, Ye J, Moller A-B, Zhang J, Gülmezoglu AM, Torloni MR (2016). The increasing trend in caesarean section rates: global, regional and national estimates: 1990-2014. PLoS One.

[33] Singh U, Ueranantasun A, Kuning M (2017). Factors associated with low birth weight in Nepal using multiple imputation. BMC Pregnancy Childbirth..

[34] Sharma SR, Giri S, Timalsina U, Bhandari SS, Basyal B, Wagle K (2015). Low birth weight at term and its determinants in a tertiary hospital of Nepal: a case-control study. PLoS One.

[35] Ministry of Health Nepal, New ERA, and ICF (2017). Nepal Demographic and Health Survery 2016: Key indicators report.

