# Supplementary material for: Domestic violence and perinatal outcomes – a prospective cohort study from Nepal
Source: BMC Public Health. 2019 May 31;19:671. doi: 10.1186/s12889-019-6967-y (PMC6545012; doi:10.1186/s12889-019-6967-y)
Supplement: Supplementary file 1 — Characteristics of women with and without delivery records in Nepal, 2016. (DOCX 25 kb) [file 12889_2019_6967_MOESM1_ESM.docx]

| **Additional file 1**. Characteristics of women with and without delivery records in Nepal, 2016. | | | | | | | |
| --- | --- | --- | --- | --- | --- | --- | --- |
| **Characteristics** | **Total** | | **Without delivery records*^a^*** | | **With delivery records*^b^*** | |  |
|  | **N=2003** | | **n=622** | | **n=1381** | |  |
|  | **n** | **%** | **n** | **%** | **n** | **%** | **p-value** |
| **Domestic violence (DV)** |  |  |  |  |  |  | 0.263 |
| No | 1582 | 79.0 | 484 | 77.8 | 1098 | 79.5 |  |
| Fear only | 251 | 12.5 | 75 | 12.0 | 176 | 12.7 |  |
| Violence only | 72 | 3.6 | 24 | 3.9 | 48 | 3.5 |  |
| Both fear and violence | 98 | 4.9 | 39 | 6.3 | 59 | 4.3 |  |
| **Any form of DV** | |  |  |  |  |  | 0.389 |
| No | 1582 | 79.0 | 484 | 77.8 | 1098 | 79.5 |  |
| Yes | 421 | 21.0 | 138 | 22.2 | 283 | 20.5 |  |
| **Sociodemographic** | |  |  |  |  |  |  |
| **Woman’s age in years [mean (SD)]** | 24.90 (4.0) | | 24.59 (4.0) | | 25.04 (4.0) | |  |
| **(n=2003)** |  |  |  |  |  |  | 0.049 |
| 15-19 | 125 | 6.2 | 48 | 7.7 | 77 | 5.6 |  |
| 20-24 | 880 | 43.9 | 289 | 46.5 | 591 | 42.8 |  |
| 25-29 | 720 | 35.9 | 201 | 32.3 | 519 | 37.6 |  |
| ≥ 30 | 278 | 13.9 | 84 | 13.5 | 194 | 14.0 |  |
| **Family structure (n=1941)** | | |  |  |  |  | 0.225 |
| Nuclear | 939 | 48.4 | 307 | 50.4 | 632 | 47.4 |  |
| Extended | 1002 | 51.6 | 302 | 49.6 | 700 | 51.6 |  |
| **Geographical setting (n=2003)** | | | |  |  |  |  |
| Rural | 627 | 31.3 | 223 | 35.8 | 404 | 29.3 | 0.003 |
| Urban | 1376 | 68.7 | 399 | 64.1 | 977 | 70.7 |  |
| **Caste and ethnicity (n=1995)** | | |  |  |  |  | 0.239 |
| Dalit***^c^*** | 57 | 2.9 | 19 | 3.1 | 38 | 2.8 |  |
| Disadvantaged Janajati***^d^*** | 450 | 22.6 | 152 | 24.7 | 298 | 21.6 |  |
| Advantaged Janajati***^e^*** | 422 | 21.2 | 116 | 18.9 | 306 | 22.2 |  |
| Upper caste***^f^*** | 1066 | 53.4 | 328 | 53.3 | 738 | 53.5 |  |
| **Woman’s education (n=1998)** | | |  |  |  |  | 0.011 |
| None | 217 | 10.9 | 86 | 13.9 | 131 | 9.5 |  |
| Primary | 288 | 14.4 | 98 | 15.8 | 190 | 13.8 |  |
| Secondary | 446 | 22.3 | 129 | 20.8 | 317 | 23.0 |  |
| Higher | 1047 | 52.4 | 306 | 49.4 | 741 | 53.7 |  |
| **Woman’s income** | |  |  |  |  |  | 0.760 |
| No income | 1488 | 74.3 | 467 | 75.1 | 1021 | 73.9 |  |
| Income no autonomy | 117 | 5.8 | 33 | 5.3 | 84 | 6.1 |  |
| Income and autonomy | 398 | 19.9 | 122 | 19.6 | 276 | 20.0 |  |
| **Knowledge and access of incentive (n=2001)** | | | | |  |  | 0.020 |
| No knowledge | 540 | 27.0 | 179 | 28.8 | 361 | 26.2 |  |
| Knowledge no access | 150 | 7.5 | 59 | 9.5 | 91 | 6.6 |  |
| Knowledge and access | 1311 | 65.5 | 384 | 61.7 | 927 | 67.2 |  |
| **Study site** |  |  |  |  |  |  | 0.256 |
| Dhulikhel Hospital (DH) | 1011 | 50.5 | 330 | 53.1 | 681 | 49.3 |  |
| Kathmandu Medical College (KMC) | 992 | 49.5 | 292 | 46.9 | 700 | 50.7 |  |
| **Baseline anxiety and depression (SCL-5 score*^g^*)** | | | | | | | 0.439 |
| ≤ 2 | 1529 | 76.3 | 468 | 75.2 | 1061 | 76.8 |  |
| > 2 | 474 | 23.7 | 154 | 24.8 | 320 | 23.2 |  |
| **Parity** |  |  |  |  |  |  | 0.057 |
| Nullipara | 994 | 49.6 | 289 | 46.5 | 705 | 51.0 |  |
| Multipara | 1009 | 50.4 | 333 | 53.5 | 676 | 49.0 |  |
| **DV and acceptance of DV (DH only n=1011)** | | | | | | | 0.256 |
| No | 686 | 67.9 | 216 | 65.5 | 470 | 69.0 |  |
| Yes | 325 | 32.1 | 114 | 34.5 | 211 | 31.0 |  |
| ***^a^***Without delivery records: women who were in the baseline study but did not deliver at two hospitals (Dhulikhel hospital and Kathmandu medical college) | | | | | | | |
| ***^b^***With delivery records: women who were in the baseline study and delivered at DH and KMC | | | | | | | |
| ***^c^***Dalit = The most oppressed social class | | | | | | | |
| ***^d^***Disadvantaged Janajati = Indigenous groups with little or no social mobility | | | | | | | |
| ***^e^***Advantaged Janajati = Indigenous groups with opportunity and access to social mobility | | | | | | | |
| ***^f^***Upper castes = Traditionally, the most privileged groups in the social hierarchy | | | | | | | |
| ***^g^***SCL-5 score: Hopkins Symptom Checklist score | | | | | | | |

|  | | | | | | | | |
| --- | --- | --- | --- | --- | --- | --- | --- | --- |
| **Additional file 2**. Distribution of obstetric characteristics among women of Nepal, 2016. | | | | | | | | |
| **Characteristics** | | **Total** | | **Dhulikhel Hospital** | | **Kathmandu Medical College** | |  |
|  |  | **N=1381** | | **n=681** | | **n=700** | |  |
|  |  | **n** | **%** | **n** | **%** | **n** | **%** | **p-value** |
| **Parity** | |  |  |  |  |  |  |  |
| Primiparous | | 705 | 51.0 | 349 | 51.2 | 356 | 50.9 | 0.914 |
| Multiparous | | 676 | 49.0 | 332 | 48.8 | 344 | 49.1 |  |
| **Antenatal visits before birth (n=1364)** | | | |  |  |  |  | <0.001 |
| < 4 |  | 294 | 21.3 | 33 | 4.9 | 261 | 38.2 |  |
| ≥ 4 |  | 1070 | 77.5 | 647 | 95.1 | 423 | 61.8 |  |
| **Mode of delivery (n=1380)** | | | |  |  |  |  | <0.001 |
| Vaginal delivery | | 809 | 54.6 | 458 | 67.3 | 351 | 50.2 |  |
| Instrumental delivery | | 52 | 3.8 | 30 | 4.4 | 22 | 3.1 |  |
| **Cesarean section (CS)** | | 519 | 37.6 | 193 | 28.3 | 326 | 46.6 |  |
|  | Cesarean section (n=518) | | |  |  |  |  | 0.030 |
|  | Elective | 79 | 15.3 | 38 | 19.7 | 41 | 12.6 |  |
|  | Emergency | 439 | 84.7 | 155 | 80.3 | 284 | 87.4 |  |
|  | Indication of Cesarean section (n=388) | | | |  |  |  | <0.001 |
|  | Prolonged labor | 59 | 15.2 | 11 | 6.4 | 48 | 22.1 |  |
|  | Breech presentation | 45 | 11.6 | 18 | 10.5 | 27 | 12.4 |  |
|  | Cephalo-pelvic disproportion (CPD) | 20 | 5.2 | 6 | 3,5 | 14 | 6.5 |  |
|  | Other fetal causes | 73 | 18.8 | 19 | 11,1 | 54 | 24.9 |  |
|  | Maternal causes | 69 | 17.8 | 63 | 36,8 | 6 | 2.8 |  |
|  | Previous cesarean section | 107 | 27.6 | 54 | 31,6 | 53 | 24.4 |  |
|  | Unknown reasons | 15 | 3.9 | 0 | 0,0 | 15 | 6.9 |  |
| **Gestational age at birth (n=1372)** | | | |  |  |  |  | 0.215 |
| < 37 weeks | | 122 | 8.9 | 67 | 9.9 | 55 | 7.9 |  |
| ≥ 37 weeks | | 1250 | 91.1 | 613 | 90.1 | 637 | 92.1 |  |
| **Mean birthweight in grams,** **mean (SD)** | | 2934.4 (474.4) | | 2900.29 (473.0) | | 2968.8 (473.6) | |  |
| **Birthweight (n=1353)** | | |  |  |  |  |  | 0.008 |
| ≥ 2500 g | | 1171 | 86.5 | 571 | 84.1 | 600 | 89.0 |  |
| < 2500 g | | 182 | 13.5 | 108 | 15.9 | 74 | 11.0 |  |
| **Live birth** | |  |  |  |  |  |  |  |
| No |  | 11 | 0.8 | 4 | 0.6 | 7 | 1.0 | 0.388 |
| Yes |  | 1370 | 99.2 | 677 | 99.4 | 693 | 99.0 |  |
| **Apgar score at five minutes after birth (n=1327)** | | | |  |  |  |  |  |
| < 7 |  | 61 | 4.6 | 42 | 6.4 | 19 | 2.8 | 0.002 |
| ≥ 7 |  | 1266 | 95.4 | 613 | 93.6 | 653 | 97.2 |  |
| **Admission to neonatal intensive care unit (n=1378)** | | | | | |  |  |  |
| No |  | 1067 | 77.4 | 658 | 96.6 | 409 | 58.7 | <0.001 |
| Yes |  | 311 | 22.6 | 23 | 3.4 | 288 | 41.3 |  |
