# Supplementary material for: Domestic violence and perinatal outcomes – a prospective cohort study from Nepal
Source: BMC Public Health. 2019 May 31;19:671. doi: 10.1186/s12889-019-6967-y (PMC6545012; doi:10.1186/s12889-019-6967-y)
Supplement: Supplementary file 2 — Distribution of obstetric characteristics among women of Nepal, 2016. (DOCX 21 kb) [file 12889_2019_6967_MOESM2_ESM.docx]

| **Additional file 2**. Distribution of obstetric characteristics among women of Nepal, 2016. | | | | | | | | |
| --- | --- | --- | --- | --- | --- | --- | --- | --- |
| **Characteristics** | | **Total** | | **Dhulikhel Hospital** | | **Kathmandu Medical College** | |  |
|  |  | **N=1381** | | **n=681** | | **n=700** | |  |
|  |  | **n** | **%** | **n** | **%** | **n** | **%** | **p-value** |
| **Parity** | |  |  |  |  |  |  |  |
| Primiparous | | 705 | 51.0 | 349 | 51.2 | 356 | 50.9 | 0.914 |
| Multiparous | | 676 | 49.0 | 332 | 48.8 | 344 | 49.1 |  |
| **Antenatal visits before birth (n=1364)** | | | |  |  |  |  | <0.001 |
| < 4 |  | 294 | 21.3 | 33 | 4.9 | 261 | 38.2 |  |
| ≥ 4 |  | 1070 | 77.5 | 647 | 95.1 | 423 | 61.8 |  |
| **Mode of delivery (n=1380)** | | | |  |  |  |  | <0.001 |
| Vaginal delivery | | 809 | 54.6 | 458 | 67.3 | 351 | 50.2 |  |
| Instrumental delivery | | 52 | 3.8 | 30 | 4.4 | 22 | 3.1 |  |
| **Cesarean section (CS)** | | 519 | 37.6 | 193 | 28.3 | 326 | 46.6 |  |
|  | Cesarean section (n=518) | | |  |  |  |  | 0.030 |
|  | Elective | 79 | 15.3 | 38 | 19.7 | 41 | 12.6 |  |
|  | Emergency | 439 | 84.7 | 155 | 80.3 | 284 | 87.4 |  |
|  | Indication of Cesarean section (n=388) | | | |  |  |  | <0.001 |
|  | Prolonged labor | 59 | 15.2 | 11 | 6.4 | 48 | 22.1 |  |
|  | Breech presentation | 45 | 11.6 | 18 | 10.5 | 27 | 12.4 |  |
|  | Cephalo-pelvic disproportion (CPD) | 20 | 5.2 | 6 | 3,5 | 14 | 6.5 |  |
|  | Other fetal causes | 73 | 18.8 | 19 | 11,1 | 54 | 24.9 |  |
|  | Maternal causes | 69 | 17.8 | 63 | 36,8 | 6 | 2.8 |  |
|  | Previous cesarean section | 107 | 27.6 | 54 | 31,6 | 53 | 24.4 |  |
|  | Unknown reasons | 15 | 3.9 | 0 | 0,0 | 15 | 6.9 |  |
| **Gestational age at birth (n=1372)** | | | |  |  |  |  | 0.215 |
| < 37 weeks | | 122 | 8.9 | 67 | 9.9 | 55 | 7.9 |  |
| ≥ 37 weeks | | 1250 | 91.1 | 613 | 90.1 | 637 | 92.1 |  |
| **Mean birthweight in grams,** **mean (SD)** | | 2934.4 (474.4) | | 2900.29 (473.0) | | 2968.8 (473.6) | |  |
| **Birthweight (n=1353)** | | |  |  |  |  |  | 0.008 |
| ≥ 2500 g | | 1171 | 86.5 | 571 | 84.1 | 600 | 89.0 |  |
| < 2500 g | | 182 | 13.5 | 108 | 15.9 | 74 | 11.0 |  |
| **Live birth** | |  |  |  |  |  |  |  |
| No |  | 11 | 0.8 | 4 | 0.6 | 7 | 1.0 | 0.388 |
| Yes |  | 1370 | 99.2 | 677 | 99.4 | 693 | 99.0 |  |
| **Apgar score at five minutes after birth (n=1327)** | | | |  |  |  |  |  |
| < 7 |  | 61 | 4.6 | 42 | 6.4 | 19 | 2.8 | 0.002 |
| ≥ 7 |  | 1266 | 95.4 | 613 | 93.6 | 653 | 97.2 |  |
| **Admission to neonatal intensive care unit (n=1378)** | | | | | |  |  |  |
| No |  | 1067 | 77.4 | 658 | 96.6 | 409 | 58.7 | <0.001 |
| Yes |  | 311 | 22.6 | 23 | 3.4 | 288 | 41.3 |  |
