# Supplementary material for: Domestic violence and perinatal outcomes – a prospective cohort study from Nepal
Source: BMC Public Health. 2019 May 31;19:671. doi: 10.1186/s12889-019-6967-y (PMC6545012; doi:10.1186/s12889-019-6967-y)
Supplement: Supplementary file 3 — Prevalence of domestic violence and low birthweight, preterm birth or cesarean section of Nepal, 2016 (DOCX 16 kb) [file 12889_2019_6967_MOESM3_ESM.docx]

| **Additional file 3.** Prevalence of domestic violence and low birthweight, preterm birth or cesarean section of Nepal, 2016. | | | | | | | | | | | | | | | | | | |
| --- | --- | --- | --- | --- | --- | --- | --- | --- | --- | --- | --- | --- | --- | --- | --- | --- | --- | --- |
|  | |  | |  | | | |  |  | | | |  |  | | | |  |
| **Domestic violence** | | **Total** | | **Low birthweight** | | | |  | **Preterm birth** | | | |  | **Cesarean section** | | | |  |
|  |  |  | | **Yes** | | **No** | |  | **Yes** | | **No** | |  | **Yes** | | **No** | |  |
|  |  | **N=1381** | | **n=182** | | **n=1171** | | **p-value** | **n=122** | | **n=1250** | | **p-value** | **n=519** | | **n=861** | | **p-value** |
|  |  | **n** | **%** | **n** | **%** | **n** | **%** |  | **n** | **%** | **n** | **%** |  | **n** | **%** | **n** | **%** |  |
| **Domestic violence** | |  |  |  |  |  |  | 0.507 |  |  |  |  | 0.154 |  |  |  |  | 0.743 |
|  | No | 1098 | 79.5 | 142 | 78.0 | 932 | 79.6 |  | 91 | 74.6 | 998 | 79.8 |  | 417 | 80.3 | 680 | 79.0 |  |
|  | Fear only (Fear but no violence) | 176 | 12.7 | 27 | 14.8 | 146 | 12.5 |  | 17 | 13.9 | 159 | 12.7 |  | 60 | 11.6 | 116 | 13.5 |  |
|  | Violence only | 48 | 3.5 | 8 | 4.4 | 40 | 3.4 |  | 4 | 3.3 | 44 | 3.5 |  | 18 | 3.5 | 30 | 3.5 |  |
|  | Both (Fear and violence) | 59 | 4.3 | 5 | 2.7 | 53 | 4.5 |  | 10 | 8.2 | 49 | 3.9 |  | 24 | 4.6 | 35 | 4.1 |  |
| **Ever** | |  |  |  |  |  |  |  |  |  |  |  |  |  |  |  |  |  |
|  | Fear | 235 | 17 | 32 | 17.6 | 199 | 17.0 | 0.844 | 27 | 22.1 | 208 | 16.6 | 0.124 | 84 | 16.2 | 151 | 17.5 | 0.517 |
|  | Emotional or physical abuse | 72 | 5.2 | 9 | 4.9 | 62 | 5.3 | 0.844 | 11 | 9.0 | 61 | 4.9 | 0.051 | 29 | 5.6 | 43 | 5.0 | 0.631 |
| **Previous year** | |  |  |  |  |  |  |  |  |  |  |  |  |  |  |  |  |  |
|  | Physical abuse | 35 | 2.5 | 2 | 1.1 | 33 | 2.8 | 0.216 | 2 | 1.6 | 33 | 2.6 | 0.763 | 17 | 3.3 | 18 | 2.1 | 0.175 |
|  | Sexual abuse | 13 | 0.9 | 1 | 0.5 | 12 | 1.0 | 1.000 | 0 | 0.0 | 13 | 1.0 | 0.620 | 5 | 1.0 | 8 | 0.9 | 0.949 |
| **Since pregnant** | |  |  |  |  |  |  |  |  |  |  |  |  |  |  |  |  |  |
|  | Physical abuse | 21 | 1.5 | 4 | 2.2 | 17 | 1.5 | 0.513 | 3 | 2.5 | 18 | 1.4 | 0.424 | 8 | 1.5 | 13 | 1.5 | 0.963 |
| Multiple responses possible | | | | | | | | |  | | | | | | | | | |
| Fear = Afraid of anyone in the family | | | | | | | | |  | | | | | | | | | |
